# Preventive behaviors adults report using to avoid catching or spreading influenza, United States, 2015-16 influenza season

**DOI:** 10.1371/journal.pone.0195085

**Published:** 2018-03-30

**Authors:** Anup Srivastav, Tammy A. Santibanez, Peng-Jun Lu, M. Christopher Stringer, Jill A. Dever, Michael Bostwick, Marshica Stanley Kurtz, Noreen L. Qualls, Walter W. Williams

**Affiliations:** 1 Leidos Inc, Atlanta, Georgia, United States of America; 2 Immunization Services Division, National Center for Immunization and Respiratory Diseases, Centers for Disease Control and Prevention, Atlanta, Georgia, United States of America; 3 Demographic Statistical Methods Division, U.S. Census Bureau, Durham, North Carolina, United States of America; 4 Division for Statistical and Data Sciences, RTI International, Washington, D.C., United States of America; 5 Statistics and Operation Research, RTI International, Research Triangle Park, North Carolina, United States of America; 6 Division of Global Migration and Quarantine, National Center for Emerging and Zoonotic Infectious Diseases, Centers for Disease Control and Prevention, Atlanta, Georgia, United States of America; University of California San Diego, UNITED STATES

## Abstract

**Introduction:**

Influenza vaccination can prevent influenza and potentially serious influenza-related complications. Although the single best way to prevent influenza is annual vaccination, everyday preventive actions, including good hygiene, health, dietary, and social habits, might help, too. Several preventive measures are recommended, including: avoiding close contact with people who are sick; staying home when sick; covering your mouth and nose when coughing or sneezing; washing your hands often; avoiding touching your eyes, nose, and mouth; and practicing other good health habits like cleaning and disinfecting frequently touched surfaces, getting plenty of sleep, and drinking plenty of fluids. Understanding public acceptance and current usage of these preventive behaviors can be useful for planning both seasonal and pandemic influenza prevention campaigns. This study estimated the percentage of adults in the United States who reported practicing preventive behaviors to avoid catching or spreading influenza, and explored associations of reported behaviors with sociodemographic factors.

**Methods:**

We analyzed data from 2015 National Internet Flu Survey, a nationally representative probability-based Internet panel survey of the non-institutionalized U.S. population ≥18 years. The self-reported behaviors used to avoid catching or spreading influenza were grouped into four and three non-mutually exclusive subgroups, respectively. Weighted proportions were calculated. Multivariable logistic regression models were used to calculate adjusted prevalence differences and to determine independent associations between sociodemographic characteristics and preventive behavior subgroups.

**Results:**

Common preventive behaviors reported were: 83.2% wash hands often, 80.0% cover coughs and sneezes, 78.2% stay home if sick with a respiratory illness, 64.4% avoid people sick with a respiratory illness, 51.7% use hand sanitizers, 50.2% get treatment as soon as possible, and 49.8% report getting the influenza vaccination. Race/ethnicity, gender, age, education, income, region, receipt of influenza vaccination, and household size were associated with use of preventive behaviors after controlling for other factors.

**Conclusion:**

Many adults in the United States reported using preventive behaviors to avoid catching or spreading influenza. Though vaccination is the most important tool available to prevent influenza, the addition of preventive behaviors might play an effective role in reducing or slowing transmission of influenza and complement prevention efforts.

## Introduction

Influenza vaccination is the best way to prevent influenza and potentially serious influenza-related complications [1,2]. The Centers for Disease Control and Prevention (CDC) recommends that all persons ≥6 months of age get an influenza vaccination each influenza season [3]. Despite the recommendations, adult influenza vaccination coverage remains suboptimal [4–6] and below *Healthy People 2020* targets [7]. Although the single best way to prevent influenza is to get vaccinated each year, supplementing it with everyday preventive actions, including good hygiene, health, dietary, and social habits, might help, too. CDC and others recommend several such measures, including: avoiding close contact with people who are sick; staying home when sick; covering your mouth and nose when coughing or sneezing; washing your hands often; avoiding touching your eyes, nose, and mouth; and practicing other good health habits like cleaning and disinfecting frequently touched surfaces, getting plenty of sleep, and drinking plenty of fluids [8–13].

Previous studies reported that hand hygiene and facemasks prevented household transmission of influenza virus [14], reduced absenteeism in school children caused by influenza-like illness (ILI) or laboratory-confirmed influenza [15], and significantly reduced influenza A (H1N1) viral counts on hands [16]. Other studies [13–19] have assessed various preventive behaviors that adults adopt other than influenza vaccination (such as social distancing, use of disinfectants, dietary supplements, maintaining a healthy lifestyle and a healthy diet, getting treatment as soon as possible) to either avoid catching or spreading the influenza virus. Understanding public acceptance and current usage of these preventive behaviors can be useful for planning both seasonal and pandemic influenza prevention campaigns.

This study uses data from the 2015 National Internet Flu Survey (NIFS) to estimate the percentage of adults in the United States who say they practiced preventive behaviors, and to explore associations of reported behaviors with sociodemographic factors. The results could be used to prepare targeted and more effective policies and risk communications for the general public to help prevent catching or spreading influenza, supplementing preexisting vaccination policies and outreach strategies. The information also can be used for modelling the spread of influenza, especially during seasons when the vaccine is not a good match to circulating influenza viruses and everyday preventive actions might be promoted more heavily.

## Materials and methods

### Survey description

The NIFS is a nationally representative probability-based Internet panel survey of the non-institutionalized U.S. population ≥18 years, sponsored by CDC. The 2015 NIFS was conducted by RTI International and GfK Custom Research, LLC. The primary goal of NIFS is to rapidly collect influenza vaccination-related data early in the influenza season [20]. The NIFS sample was drawn from GfK’s KnowledgePanel^®^ [21], an online panel consisting of a representative random sample of the U.S. population. The KnowledgePanel^®^ recruitment response rate was approximately 13% using the American Association for Public Opinion Research response rate 3 formula [22]. The survey was conducted in English only. For the GfK panel, participants were initially chosen by a random selection of residential addresses and were continuously recruited. Persons in selected households were then invited to become a member of the survey panel. For those who agreed to participate but did not already have Internet access, GfK provided both a computer and Internet access at no cost. Panelists received unique login information for accessing surveys online, and were sent e-mails inviting them to participate in a variety of surveys.

The 2015 NIFS sampling design was a single-stage stratified sample with oversampling of select subgroups of particular analytical interest. Twelve mutually exclusive design strata were defined as the interaction of two categorical variables—*age* (18–49 years, 50–64 years, and 65 years and older) and *race/ethnicity* (Hispanic, non-Hispanic white, non-Hispanic black, and non-Hispanic other/multiple races)—known for all members of the probability-based Internet panel. Independent random samples were selected within each design stratum. A total of 6,148 panel members across the 12 design strata were randomly sampled using probabilities of selection inversely proportional to the KnowledgePanel^®^ survey weight (a base weight adjusted for nonresponse) from 42,075 eligible panelists, with a target of 4,025 completed surveys. A total of 3,301 completed the survey, with a completion rate of 53.7% (unweighted). Sample members were offered the standard KnowledgePanel^®^ incentive—1,000 points (equivalent to $1); they were not offered additional incentives for NIFS participation specifically. The NIFS was conducted from October 29 to November 11, 2015.

The NIFS collects information about early-season influenza vaccination and knowledge, attitudes, and behaviors related to influenza and influenza vaccination in the U.S. adult population. Additionally, other information such as demographic and access-to-care characteristics was collected. In the 2015 NIFS, new questions regarding precautions taken to avoid catching influenza or spreading influenza to others were added to the survey.

### Preventive behavior assessment

Information on the preventive behaviors among adults aged ≥18 years were based on two survey questions. To determine precautions taken to avoid catching influenza, respondents were asked: *In general*, *what precautions do you take to avoid catching the flu*? *Check all that apply*. The response options were: getting a flu vaccination; washing your hands often; using hand sanitizers; avoiding people who are sick with a respiratory illness; covering your mouth and nose with a mask; taking vitamins; taking herbal medicine or products; other (specify); or none of the above. To determine precautions taken to avoid spreading influenza to others, respondents were asked: *In general*, *if you get the flu*, *what precautions do you take to avoid passing the flu to others*? *Check all that apply*. The response options were: washing your hands often; using hand sanitizers; staying home if sick with a respiratory illness; covering your mouth and nose with a mask; covering coughs and sneezes; getting treatment as soon as possible; other (specify); or none of the above. The verbatim responses were evaluated and back-coded into existing categories or newly created categories, resulting in 14 behaviors to avoid catching influenza and 12 behaviors to avoid spreading influenza to others included in this analysis.

For succinctness, the resulting 14 behaviors to avoid catching influenza were grouped into four non-mutually exclusive groups (a person could be in multiple groups). These groups were: 1) getting an influenza vaccination; 2) personal hygiene behaviors (included washing hands often, using hand sanitizers, and using disinfectants); 3) personal health and dietary behaviors (included maintaining a healthy diet, getting regular exercise, getting adequate rest, maintaining a healthy lifestyle, taking vitamins, taking herbal medicine or products, and drinking ample fluids); and/or 4) interpersonal social behaviors (included avoiding people who are sick with a respiratory illness, covering mouth and nose with a mask, and avoiding others in general). Likewise, the 12 behaviors to avoid spreading influenza to others were grouped into three non-mutually exclusive groups. These groups were: 1) personal hygiene behaviors (included washing hands often and using hand sanitizers); 2) personal health and dietary behaviors (included getting adequate rest, getting treatment as soon as possible, drinking ample fluids, and taking supplements); and/or 3) interpersonal social behaviors (included staying home if sick with a respiratory illness, covering your mouth and nose with a mask, covering coughs and sneezes, avoiding others in general, and using disinfectants). A person could be included in more than one of these groupings.

### Influenza vaccination and other variables

Other sociodemographic variables collected during the NIFS or as part of GfK panel recruitment included age, gender, race/ethnicity, marital status, educational level, employment status, annual household income, region of residence, metropolitan statistical area (MSA) status, and household size. Receipt of influenza vaccination (as of the date respondents completed the online survey) also is reported based upon the survey question, *A flu vaccination can be a shot injected in the arm or a mist sprayed in the nose by a doctor*, *nurse*, *pharmacist or other health professional*. *Since July 1*, *2015*, *have you had a flu vaccination*? Influenza vaccination coverage estimates represent the cumulative proportion of persons vaccinated by the time when the survey was completed [20].

### Data analysis

Unadjusted proportions are reported along with 95% confidence intervals. All analyses were weighted to the U.S. population of non-institutionalized adults using the 2015 March Supplement of the Current Population Survey estimates after adjusting the NIFS base weights for nonresponse. A bias analysis was conducted to determine the influence of nonresponse in the results using demographic and geographic information available in the KnowledgePanel^®^ sampling frame. Tests were performed with a significance level set at α = 0.05. Multivariable logistic regression was conducted to calculate adjusted prevalence differences (adjusting for all variables included in the model) and to determine the independent associations between sociodemographic characteristics and preventive behavior subgroups. Separate regression models were run for each preventive behavior subgroup because the groupings were not mutually exclusive. T-tests were used to test differences in adjusted coverage within each covariate category compared with appropriate reference groups for each preventive behavior subgroup. All analyses were performed using SAS, release 9.4 (SAS Inc. Cary, NC, USA) and SUDAAN, release 11.0.1 (Research Triangle Institute, Research Triangle Park, NC, USA).

The NIFS was designated as “Public Health Non-Research” during the determination for applicability of human subjects’ regulations, because the activity is not intended to include applicable research, but to access the implementation, coverage, performance, and/or satisfaction with an existing public health program, service, function, intervention or recommendation. Data security was addressed and informed consent was sought.

## Results

The sociodemographic characteristics of the study population are presented in Table 1. The majority of respondents were aged 18–49 years (54.7%), female (51.7%), of non-Hispanic white race ethnicity (65.5%), were married or living with a partner (57.9%), had high school or higher education (88.0%), were employed (58.5%), lived in a metropolitan statistical area (83.5%), and lived in households of at least two or more (88.8%) (Table 1).

**Table 1:** Sociodemographic characteristics among adults aged ≥18 years–United States, National Internet Flu Survey 2015.

Characteristic	Unweighted Sample Size, No.	Weighted Percentage % (95% CI)
Total	3,301	100.0
**Age**		
18–49 years	1,508	54.7 (53.8, 55.6)
50–64 years	1,033	26.0 (25.3, 26.7)
≥65 years	760	19.3 (18.6, 19.9)
**Gender**		
Male	1,622	48.3 (46.3, 50.4)
Female	1,679	51.7 (49.6, 53.7)
**Race/ethnicity**		
Non-Hispanic white only	1,922	65.5 (64.3, 66.7)
Non-Hispanic black only	494	11.8 (11.0, 12.6)
Hispanic	460	14.1 (13.0, 15.3)
Non-Hispanic, other or multiple races	425	8.6 (7.7, 9.5)
**Marital status**		
Married/living with partner	1,950	57.9 (55.9, 59.9)
Widowed/divorced/separated	616	17.7 (16.2, 19.2)
Never married	735	24.5 (22.7, 26.3)
**Education level**		
Less than high school	269	12.0 (10.5, 13.6)
High school	892	29.4 (27.6, 31.3)
Some college	956	28.5 (26.8, 30.4)
Bachelor's degree or higher	1,184	30.1 (28.4, 31.8)
**Employment**		
Employed	1,862	58.5 (56.6, 60.3)
Unemployed	188	6.3 (5.3, 7.5)
Not in work force	1,251	35.2 (33.5, 36.9)
**Annual household income**		
<$35,000	945	27.2 (25.5, 29.1)
$35,000-$49,999	374	11.6 (10.3, 13.0)
$50,000-$74,999	628	17.9 (16.4, 19.5)
≥$75,000	1,354	43.3 (41.3, 45.3)
**Region of residence**		
Northeast	590	18.1 (16.6, 19.7)
Midwest	693	21.4 (19.8, 23.1)
South	1,185	37.1 (35.1, 39.0)
West	833	23.4 (21.8, 25.1)
**MSA status**		
Metro	2,885	85.0 (83.5, 86.4)
Non-metro	416	15.0 (13.6, 16.5)
**Household size**		
One	723	21.2 (19.6, 22.9)
Two	1,272	36.1 (34.3, 37.9)
Three or more	1,306	42.7 (40.8, 44.7)
**Received influenza vaccination**		
Yes	1,354	39.9 (38.0, 41.8)
No	1,894	60.1 (58.2, 62.0)

**Fig 1:**
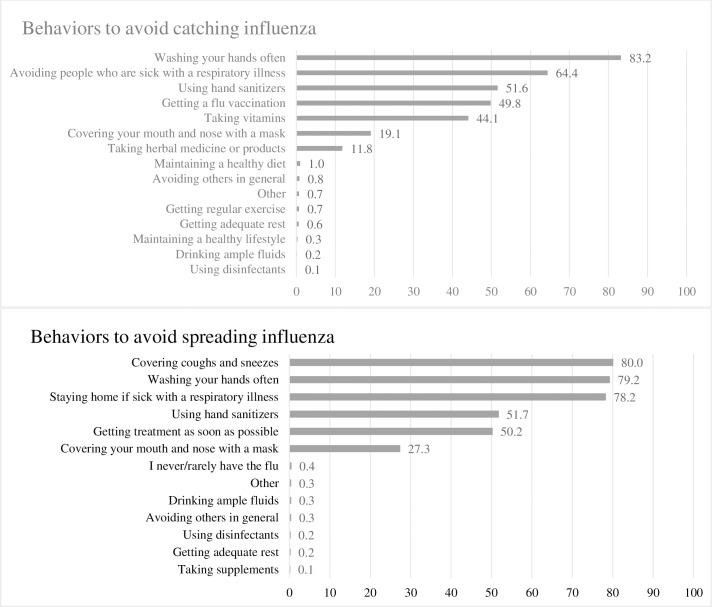
Preventive behaviors used by adults as precautions taken to avoid catching influenza or spreading influenza.

Among adults aged ≥18 years, 39.9% reported having received influenza vaccination by early November (Table 1). The percentages of adults reporting each preventative behavior to avoid catching influenza and spreading influenza to others are presented in Fig 1. The most commonly reported behaviors for avoiding catching influenza were: washing hands often (83.2%), avoiding people who are sick with a respiratory illness (64.4%), using hand sanitizers (51.6%), getting an influenza vaccination (49.8%), taking vitamins (44.1%), covering mouth and nose with a mask (19.1%), and taking herbal medicine or products (11.8%) (Fig 1). The most commonly reported behaviors for avoiding spreading influenza to others were: covering coughs and sneezes (80.0%), washing hands often (79.2%), staying home if sick with a respiratory illness (78.2%), using hand sanitizers (51.7%), getting treatment as soon as possible (50.2%), and covering mouth and nose with a mask (27.3%) (Fig 1).

Getting an influenza vaccination was not the most common preventive behavior reported by respondents. The most common preventive behaviors and approaches reported were personal hygiene behaviors (84.9%), followed by interpersonal social behaviors (68.0%), influenza vaccination (49.8%), and personal health and dietary behaviors (47.1%) (Table 2). The most common behaviors to avoid spreading influenza reported were interpersonal social behaviors (89.5%), followed by personal hygiene behaviors (81.1%) and personal health and dietary behaviors (50.5%). In the bivariate analyses, characteristics associated with more frequent adoption of behaviors to avoid catching or spreading influenza included, age ≥50 years, being female, non-Hispanic white or non-Hispanic other or multiple races, being married/living with partner or widowed/divorced/separated, having some college or bachelor’s degree or higher education, having annual household income ≥$35,000, and having received influenza vaccination (Table 2).

**Table 2:** Unadjusted preventive behaviors used by adults to avoid catching or spreading influenza by sociodemographic characteristics.

	Behaviors Adopted to Avoid Catching Influenza				Behaviors Adopted to Avoid Spreading Influenza		
	Getting an Influenza Vaccination	Personal Hygiene Behaviors^a^	Personal Health and Dietary Behaviors^b^	Interpersonal Social Behaviors^c^	Personal Hygiene Behaviors^a^	Personal Health and Dietary Behaviors^b^	Interpersonal Social Behaviors^c^
Characteristic	% (95% CI)	% (95% CI)	% (95% CI)	% (95% CI)	% (95% CI)	% (95% CI)	% (95% CI)
**Overall**	49.8 (47.8, 51.8)	84.9 (83.3, 86.4)	47.1 (45.1, 49.1)	68.0 (66.0, 69.9)	81.1 (79.3, 82.7)	50.5 (48.5, 52.5)	89.5 (88.0, 90.8)
**Age**							
18–49 years^d^	40.4 (37.5, 43.4)	82.4 (79.7, 84.7)	44.9 (42.0, 47.9)	65.0 (62.0, 67.9)	79.7 (76.9, 82.1)	46.6 (43.6, 49.6)	86.6 (84.3, 88.6)
50–64 years	53.5* (50.0, 56.9)	87.1* (84.4, 89.4)	47.4 (44.0, 50.8)	69.2 (65.8, 72.3)	82.6 (79.8, 85.2)	50.7 (47.2, 54.1)	90.7* (88.1, 92.7)
≥65 years	71.5* (67.7, 75.0)	89.1* (86.6, 91.2)	52.8* (48.8, 56.8)	74.7* (71.0, 78.1)	83.0 (79.9, 85.6)	61.6* (57.6, 65.4)	96.0* (94.1, 97.2)
**Gender**							
Male^d^	46.4 (43.6, 49.2)	79.3 (76.6, 81.7)	41.5 (38.7, 44.3)	64.6 (61.8, 67.4)	74.7 (72.0, 77.3)	46.4 (43.6, 49.3)	85.7 (83.3, 87.8)
Female	53.0* (50.2, 55.8)	90.2* (88.1, 91.9)	52.3* (49.5, 55.2)	71.1* (68.4, 73.6)	87.0* (84.8, 88.9)	54.3* (51.5, 57.2)	93.0* (91.3, 94.4)
**Race/ethnicity**							
Non-Hispanic white only^d^	52.8 (50.4, 55.2)	86.2 (84.3, 87.9)	47.1 (44.6, 49.5)	69.6 (67.2, 71.8)	82.2 (80.1, 84.0)	49.7 (47.2, 52.1)	91.1 (89.4, 92.5)
Non-Hispanic black only	41.5* (36.3, 46.8)	83.5 (78.6, 87.4)	46.3 (40.9, 51.8)	63.5* (57.9, 68.8)	79.1 (73.8, 83.6)	56.9* (51.3, 62.3)	86.7* (82.1, 90.2)
Hispanic	45.1* (39.1, 51.2)	82.2 (76.3, 86.8)	46.4 (40.4, 52.6)	62.8* (56.5, 68.8)	80.6 (74.7, 85.4)	50.9 (44.8, 57.0)	86.0* (80.8, 89.9)
Non-Hispanic, other or multiple races	46.2 (39.7, 52.9)	81.5 (74.3, 87.0)	49.3 (42.6, 55.9)	70.2 (63.5, 76.2)	76.3 (69.1, 82.2)	47.5 (40.9, 54.2)	86.7 (80.7, 91.1)
**Marital status**							
Married/living with partner^d^	53.4 (50.8, 55.9)	87.7 (85.7, 89.4)	49.7 (47.1, 52.3)	70.3 (67.9, 72.7)	84.0 (81.9, 85.9)	53.0 (50.4, 55.5)	91.5 (89.8, 93.0)
Widowed/divorced/separated	54.4 (49.6, 59.0)	87.2 (83.5, 90.2)	47.2 (42.5, 51.9)	69.9 (65.2, 74.3)	80.3 (75.9, 84.0)	55.7 (50.9, 60.3)	91.5 (88.3, 93.9)
Never married	38.1* (33.9, 42.4)	76.7* (72.6, 80.4)	40.9* (36.7, 45.2)	60.9* (56.5, 65.2)	74.7* (70.6, 78.5)	41.0* (36.8, 45.4)	83.1* (79.4, 86.4)
**Education level**							
Less than high school^d^	43.5 (36.6, 50.6)	73.4 (66.2, 79.5)	41.9 (35.0, 49.1)	61.3 (54.0, 68.2)	71.8 (64.7, 77.9)	49.8 (42.7, 57.0)	81.5 (75.2, 86.5)
High school	47.4 (43.6, 51.1)	80.5 (77.1, 83.5)	44.3 (40.6, 48.1)	65.7 (61.9, 69.3)	77.8 (74.3, 80.9)	48.7 (45.0, 52.5)	87.2 (84.3, 89.7)
Some college	46.1 (42.4, 49.8)	89.8* (87.2, 92.0)	50.4* (46.6, 54.2)	70.9* (67.4, 74.3)	85.6* (82.6, 88.1)	54.9 (51.1, 58.6)	91.1* (88.4, 93.1)
Bachelor's degree or higher	58.2* (55.0, 61.5)	89.2* (86.9, 91.1)	48.7 (45.5, 52.0)	70.0* (66.9, 73.0)	83.7* (80.9, 86.2)	48.4 (45.1, 51.6)	93.3* (91.3, 94.9)
**Employment**							
Employed^d^	45.2 (42.6, 47.8)	85.8 (83.6, 87.7)	46.4 (43.7, 49.0)	66.2 (63.6, 68.7)	82.1 (79.8, 84.2)	47.2 (44.6, 49.9)	88.9 (87.0, 90.6)
Unemployed	39.1 (30.8, 48.1)	78.4 (69.6, 85.2)	35.6* (27.8, 44.2)	61.1 (51.8, 69.7)	76.1 (67.0, 83.4)	46.4 (37.6, 55.4)	84.0 (75.8, 89.8)
Not in work force	59.4* (56.2, 62.6)	84.7 (81.9, 87.1)	50.4 (47.1, 53.6)	72.1* (68.9, 75.1)	80.3 (77.4, 82.9)	56.7* (53.4, 60.0)	91.4 (89.1, 93.2)
**Annual household income**							
<$35,000^d^	42.0 (38.2, 45.9)	77.9 (74.2, 81.1)	42.5 (38.7, 46.4)	62.7 (58.7, 66.6)	75.0 (71.3, 78.4)	47.5 (43.6, 51.4)	81.7 (78.1, 84.8)
$35,000-$49,999	46.3 (40.4, 52.3)	85.0* (79.5, 89.3)	47.9 (41.9, 54.0)	71.6* (65.8, 76.7)	82.5* (76.9, 87.0)	50.3 (44.3, 56.4)	90.1* (85.8, 93.2)
$50,000-$74,999	50.5* (45.8, 55.1)	88.7* (85.2, 91.5)	50.4* (45.8, 55.1)	66.1 (61.4, 70.5)	83.9* (79.8, 87.3)	48.8 (44.2, 53.5)	93.7* (90.6, 95.8)
≥$75,000	55.4* (52.3, 58.4)	87.7* (85.3, 89.8)	48.3* (45.3, 51.4)	71.1* (68.1, 73.8)	83.3* (80.7, 85.6)	53.2* (50.1, 56.2)	92.5* (90.5, 94.1)
**Region of residence**							
Northeast^d^	52.2 (47.5, 56.9)	85.5 (81.9, 88.6)	47.2 (42.5, 51.9)	62.9 (58.2, 67.4)	80.3 (76.1, 83.9)	55.1 (50.3, 59.7)	88.3 (84.7, 91.2)
Midwest	52.3 (48.0, 56.5)	86.1 (82.6, 89.0)	47.5 (43.2, 51.8)	67.3 (63.0, 71.2)	83.4 (79.9, 86.5)	44.5* (40.3, 48.7)	91.8 (88.8, 94.0)
South	50.8 (47.4, 54.1)	83.4 (80.4, 86.1)	46.4 (43.1, 49.8)	71.3* (68.1, 74.4)	81.1 (78.1, 83.8)	55.3 (51.9, 58.7)	88.4 (85.8, 90.6)
West	44.2* (40.2, 48.3)	85.7 (82.1, 88.6)	47.7 (43.6, 51.9)	67.1 (63.0, 71.0)	79.4 (75.5, 82.8)	44.9* (40.8, 49.1)	89.9 (86.8, 92.4)
**Received influenza vaccination**							
Yes	89.5* (87.2, 91.4)	89.1* (86.8, 91.0)	46.2 (43.1, 49.3)	69.8 (66.8, 72.7)	85.7* (83.2, 87.8)	55.6* (52.5, 58.7)	93.2* (91.4, 94.7)
No^d^	24.7 (22.6, 27.0)	83.9 (81.7, 86.0)	49.0 (46.3, 51.7)	68.5 (65.9, 71.0)	79.8 (77.4, 82.0)	48.3 (45.6, 51.0)	88.9 (86.9, 90.6)
**MSA status**							
Metro	50.4 (48.3, 52.6)	85.1 (83.4, 86.8)	46.8 (44.7, 49.0)	68.3 (66.2, 70.3)	81.1 (79.2, 82.9)	50.6 (48.4, 52.8)	89.5 (87.9, 90.8)
Non-metro^d^	46.2 (40.9, 51.7)	83.6 (78.9, 87.5)	48.4 (43.0, 53.9)	66.0 (60.5, 71.1)	80.9 (76.2, 84.9)	50.1 (44.6, 55.5)	89.6 (85.2, 92.8)
**Household size**							
One	47.1 (42.8, 51.4)	77.8* (73.7, 81.5)	45.4 (41.1, 49.8)	62.9* (58.4, 67.2)	72.9* (68.5, 76.8)	43.3* (39.1, 47.6)	85.2* (81.5, 88.2)
Two	56.5* (53.3, 59.6)	86.0 (83.3, 88.3)	47.8 (44.7, 51.0)	70.4 (67.3, 73.3)	81.8 (78.9, 84.3)	53.0 (49.8, 56.1)	90.6 (88.0, 92.6)
Three or more^d^	45.6 (42.4, 48.8)	87.5 (85.0, 89.6)	47.3 (44.1, 50.5)	68.4 (65.3, 71.4)	84.5 (82.0, 86.8)	52.0 (48.8, 55.3)	90.7 (88.6, 92.4)

*CI* confidence interval.

*Significant at *P* < 0.05 by t-test compared to the reference group.

^a^To **avoid catching** influenza, personal hygiene behaviors is comprised of the following behaviors: a) washing your hands often; b) using hand sanitizers; c) using disinfectants.

To **avoid spreading** influenza to others, personal hygiene behaviors is comprised of the following behaviors: a) washing your hands often; b) using hand sanitizers.

^b^To **avoid catching** influenza, personal health and dietary behaviors is comprised of the following behaviors: a) maintaining a healthy diet; b) getting regular exercise; c) getting adequate rest; d) maintaining a healthy lifestyle; e) taking vitamins; f) taking herbal medicine or products; g) drinking ample fluids.

To **avoid spreading** influenza to others, personal health and dietary behaviors is comprised of the following behaviors: a) getting adequate rest; b) getting treatment as soon as possible; c) drinking ample fluids; d) taking supplements.

^c^To **avoid catching** influenza, interpersonal social behaviors is comprised of the following behaviors: a) avoiding people who are sick with a respiratory illness; b) covering your mouth and nose with a mask; c) avoiding others in general.

To **avoid spreading** influenza to others, interpersonal social behaviors is comprised of the following behaviors: a) staying home if sick with a respiratory illness; b) covering your mouth and nose with a mask; c) covering coughs and sneezes; d) avoiding others in general; e) using disinfectants.

^d^Reference level.

Only a few of the variables found to be significantly associated with the behaviors based on the bivariate analyses remained significant in the multivariable models presented in Table 3. Characteristics independently associated with a respondent reporting that getting an influenza vaccination is a behavior they use to avoid catching influenza were: age ≥50 years, being female, having annual household income ≥$75,000, not being in the workforce, and self-report that they had received an influenza vaccination by the time of the interview for the 2015–16 influenza season. Characteristics independently associated with use of any of the personal hygiene behaviors were: age ≥50 years, being female, some college and bachelor’s degree or higher education, having annual household income $50,000-$74,999, and having ≥3 persons in the household. Characteristics independently associated with use of any of the personal health and dietary behaviors were: age ≥65 years, being female, some college, and having received an influenza vaccination. Characteristics independently associated with use of any of the interpersonal social behaviors were: age ≥65 years, being female, some college, having annual household income $35,000-$49,999, and residence in the south (Table 3).

**Table 3:** Adjusted estimates of preventive behavior responses used by adults to avoid catching or spreading influenza.

	Behaviors Adopted to Avoid Catching Influenza				Behaviors Adopted to Avoid Spreading Influenza		
	Getting an Influenza Vaccination	Personal Hygiene Behaviors^a^	Personal Health and Dietary Behaviors^b^	Interpersonal Social Behaviors^c^	Personal Hygiene Behaviors^a^	Personal Health and Dietary Behaviors^b^	Interpersonal Social Behaviors^c^
Characteristic	APD^d^ (95% CI)	APD (95% CI)	APD (95% CI)	APD (95% CI)	APD (95% CI)	APD (95% CI)	APD (95% CI)
**Age**							
18–49 years	Referent	Referent	Referent	Referent	Referent	Referent	Referent
50–64 years	6.5* (2.5, 10.5)	6.6* (2.7, 10.5)	2.6 (-2.5, 7.7)	4.0 (-0.9, 8.8)	5.1* (1.0, 9.2)	3.5 (-1.6, 8.5)	4.8* (1.5, 8.1)
≥65 years	12.8* (6.6, 18.9)	8.5* (4.0, 13.0)	7.5* (0.5, 14.4)	6.9* (0.3, 13.6)	5.7* (0.5, 11.0)	12.1* (5.3, 18.9)	8.8* (5.3, 12.2)
**Gender**							
Male	Referent	Referent	Referent	Referent	Referent	Referent	Referent
Female	3.2* (0.0, 6.3)	9.6* (6.6, 12.6)	10.3* (6.2, 14.3)	4.9* (1.0, 8.8)	11.2* (8.0, 14.4)	5.3* (1.3, 9.3)	5.7* (3.1, 8.3)
**Race/ethnicity**							
Non-Hispanic white only	Referent	Referent	Referent	Referent	Referent	Referent	Referent
Non-Hispanic black only	-4.6 (-9.5, 0.4)	2.0 (-2.1, 6.1)	3.8 (-2.5, 10.1)	-3.6 (-9.7, 2.5)	0.2 (-4.9, 5.2)	9.6* (3.6, 15.7)	0.1 (-3.2, 3.5)
Hispanic	0.7 (-4.8, 6.3)	-0.0 (-4.7, 4.6)	5.2 (-1.7, 12.2)	-2.6 (-9.3, 4.1)	2.7 (-2.3, 7.7)	5.3 (-1.5, 12.1)	-0.9 (-4.7, 2.8)
Non-Hispanic, other or multiple races	-1.3 (-6.4, 3.8)	-3.1 (-8.9, 2.7)	4.9 (-2.4, 12.2)	3.5 (-3.1, 10.0)	-3.9 (-10.0, 2.3)	1.9 (-5.5, 9.4)	-2.9 (-8.0, 2.1)
**Marital status**							
Married/living with partner	Referent	Referent	Referent	Referent	Referent	Referent	Referent
Widowed/divorced/separated	1.7 (-3.2, 6.5)	2.4 (-2.4, 7.1)	-4.1 (-10.7, 2.4)	2.0 (-4.0, 8.0)	-0.2 (-5.6, 5.2)	4.8 (-1.7, 11.3)	1.9 (-1.9, 5.6)
Never married	3.0 (-1.9, 8.0)	-2.0 (-6.5, 2.6)	-5.4 (-11.8, 0.9)	-2.4 (-8.3, 3.5)	-1.3 (-6.4, 3.7)	-4.6 (-11.0, 1.7)	0.4 (-3.2, 4.1)
**Education level**							
Less than high school	Referent	Referent	Referent	Referent	Referent	Referent	Referent
High school	0.6 (-5.4, 6.7)	5.8 (-1.5, 13.0)	2.9 (-5.2, 11.1)	3.9 (-4.2, 12.1)	5.2 (-2.0, 12.4)	0.6 (-7.5, 8.8)	2.8 (-2.3, 8.0)
Some college	0.9 (-5.1, 7.0)	14.8* (7.6, 22.0)	9.3* (0.9, 17.6)	9.2* (1.1, 17.3)	12.8* (5.7, 19.9)	7.7 (-0.6, 16.0)	5.8* (0.7, 11.0)
Bachelor's degree or higher	5.1 (-1.2, 11.4)	13.7* (6.2, 21.1)	7.8 (-0.9, 16.4)	8.0 (-0.4, 16.3)	11.1* (3.7, 18.5)	0.6 (-8.0, 9.2)	7.3* (1.9, 12.6)
**Employment**							
Employed	Referent	Referent	Referent	Referent	Referent	Referent	Referent
Unemployed	3.4 (-4.2, 11.0)	-1.2 (-7.3, 4.9)	-4.3 (-13.5, 5.0)	1.9 (-7.2, 11.0)	0.5 (-5.9, 7.0)	2.7 (-6.6, 12.0)	1.9 (-2.7, 6.5)
Not in work force	5.1* (1.0, 9.1)	-2.8 (-6.9, 1.2)	2.4 (-2.8, 7.6)	4.6 (-0.4, 9.6)	-3.2 (-7.5, 1.1)	5.1 (-0.0, 10.2)	0.7 (-2.5, 3.9)
**Annual household income**							
<$35,000	Referent	Referent	Referent	Referent	Referent	Referent	Referent
$35,000-$49,999	2.5 (-2.5, 7.4)	3.3 (-2.2, 8.8)	4.7 (-2.6, 12.0)	7.0* (0.1, 13.9)	3.5 (-2.5, 9.4)	2.3 (-5.0, 9.7)	6.8* (2.3, 11.4)
$50,000-$74,999	4.5 (-0.3, 9.4)	5.0* (0.6, 9.5)	5.9 (-0.6, 12.5)	1.2 (-5.0, 7.5)	4.6 (-0.4, 9.6)	0.9 (-5.4, 7.2)	9.4* (5.2, 13.5)
≥$75,000	9.7* (4.8, 14.6)	2.6 (-2.1, 7.4)	4.0 (-2.2, 10.2)	5.2 (-0.8, 11.1)	2.4 (-2.8, 7.5)	6.5* (0.3, 12.7)	8.4* (4.2, 12.6)
**Region of residence**							
Northeast	Referent	Referent	Referent	Referent	Referent	Referent	Referent
Midwest	0.4 (-4.5, 5.2)	0.3 (-4.1, 4.6)	-0.4 (-6.7, 6.0)	4.0 (-2.1, 10.2)	2.7 (-2.2, 7.6)	-11.6* (-17.9, -5.3)	3.6 (-0.4, 7.5)
South	1.1 (-3.3, 5.5)	-1.9 (-5.9, 2.2)	-1.5 (-7.2, 4.3)	8.7* (3.1, 14.3)	1.0 (-3.6, 5.5)	-2.0 (-7.7, 3.7)	0.9 (-2.8, 4.7)
West	-3.9 (-8.6, 0.7)	0.5 (-3.8, 4.8)	0.4 (-5.9, 6.7)	3.9 (-2.2, 10.0)	-0.7 (-5.6, 4.3)	-10.7* (-17.0, -4.5)	2.2 (-1.8, 6.2)
**Received influenza vaccination**							
Yes	61.6* (58.4, 64.8)	2.9 (-0.1, 5.8)	-5.4* (-9.5, -1.2)	-1.1 (-5.1, 2.9)	4.5* (1.3, 7.7)	4.7* (0.5, 8.8)	1.9 (-0.7, 4.5)
No	Referent	Referent	Referent	Referent	Referent	Referent	Referent
**MSA status**							
Metro	2.0 (-2.4, 6.4)	-0.1 (-4.3, 4.1)	-2.7 (-8.8, 3.3)	2.6 (-3.3, 8.4)	-0.4 (-4.9, 4.1)	-1.8 (-7.7, 4.2)	-1.5 (-5.0, 2.0)
Non-metro	Referent	Referent	Referent	Referent	Referent	Referent	Referent
**Household size**							
One	-3.9 (-9.7, 1.9)	-10.4* (-16.4, -4.3)	1.2 (-5.7, 8.1)	-4.5 (-11.4, 2.3)	-10.4* (-16.9, -3.9)	-10.7* (-17.6, -3.9)	-4.7* (-9.2, -0.3)
Two	1.5 (-2.3, 5.3)	-4.4* (-7.8, -1.0)	-1.2 (-6.1, 3.8)	-1.4 (-6.0, 3.1)	-4.8* (-8.6, -1.0)	-3.3 (-8.1, 1.6)	-2.6 (-5.6, 0.3)
Three or more	Referent	Referent	Referent	Referent	Referent	Referent	Referent

*APD* Adjusted prevalence difference; *CI* confidence interval.

*Significant at *P* < 0.05 by t-test compared to the reference group.

^a^To **avoid catching** influenza, personal hygiene behaviors is comprised of the following behaviors: a) washing your hands often; b) using hand sanitizers; c) using disinfectants.

To **avoid spreading** influenza to others, personal hygiene behaviors is comprised of the following behaviors: a) washing your hands often; b) using hand sanitizers.

^b^To **avoid catching** influenza, personal health and dietary behaviors is comprised of the following behaviors: a) maintaining a healthy diet; b) getting regular exercise; c) getting adequate rest; d) maintaining a healthy lifestyle; e) taking vitamins; f) taking herbal medicine or products; g) drinking ample fluids.

To **avoid spreading** influenza to others, personal health and dietary behaviors is comprised of the following behaviors: a) getting adequate rest; b) getting treatment as soon as possible; c) drinking ample fluids; d) taking supplements.

^c^To **avoid catching** influenza, interpersonal social behaviors is comprised of the following behaviors: a) avoiding people who are sick with a respiratory illness; b) covering your mouth and nose with a mask; c) avoiding others in general.

To **avoid spreading** influenza to others, interpersonal social behaviors is comprised of the following behaviors: a) staying home if sick with a respiratory illness; b) covering your mouth and nose with a mask; c) covering coughs and sneezes; d) avoiding others in general; e) using disinfectants.

^d^Adjusted prevalence differences, adjusted for all variables included in the table.

The characteristics independently associated with a respondent reporting using any of the personal hygiene behaviors to avoid spreading influenza to others were: age ≥50 years, being female, some college or bachelor’s degree or higher education, having received an influenza vaccination, and having ≥3 persons in the household. Characteristics independently associated with use of any of the personal health and dietary behaviors were: age ≥65 years, being female, being of non-Hispanic black race/ethnicity, having income ≥$75,000, living in the northeast, having received an influenza vaccination, and having ≥2 persons in the household. Characteristics independently associated with use of any of the interpersonal social behaviors were: age ≥50 years, being female, some college or bachelor’s degree or higher education, having income ≥$35,000, and a larger household size with ≥2 persons (Table 3).

In the bias analysis to determine the influence of nonresponse in the results, tests for the smallest race/ethnicity group (non-Hispanic other/multiple category) showed the highest detectable levels of bias, most falling below substantively meaningful levels of 10 percentage points.

## Discussion

The best way to prevent seasonal influenza is to get vaccinated each year. However, good health habits like covering coughs and washing hands often can help slow the spread of influenza viruses. The findings of this study provide insight into various preventive behaviors reportedly used by adults in the United States, either to prevent getting infected with influenza or to prevent transmitting influenza to others when infected with influenza viruses.

The distribution of self-reported preventive behaviors most frequently used by U.S. adults observed in this study were somewhat similar to the distribution reported previously from a national survey during the 2009 H1N1 influenza pandemic [17] which explored racial/ethnic differences in the adoption of preventive behaviors. Similarities observed were in frequency of use of the following behaviors: getting the seasonal influenza vaccine, washing hands often, using hand sanitizer, covering coughs and sneezes, avoiding people who are sick with a respiratory illness, and taking herbal supplements. Differences observed were in the following behaviors: frequency of use of disinfectants and avoiding others in general was lower in this study than previously reported and frequency of getting treatment as soon as possible was higher in this study than previously reported.

Use of facemasks and hand washing were reported to prevent household influenza transmission when healthy family members started using these measures within 36 hours of symptom onset with an infected family member [14]; adherence to the interventions, however, was reported to be low. A study [15] reported hand hygiene behavior (e.g., washing hands twice each day) reduced ILI or laboratory-confirmed influenza in school children by 40% and 50%, respectively. Another study [18] reported a significant association of regular physical exercise, optimal hand hygiene, face mask use when going to hospitals, and not sharing towels and handkerchiefs with a lower likelihood of reporting ILI. In addition, in another report [16], using alcohol-based hand sanitizers significantly reduced H1N1 viral counts on hands. These studies [14–16,18] provided scientific evidence that facemasks and hand hygiene behaviors are effective in reducing the transmission of influenza and ILI, suggesting that nonpharmaceutical interventions play important roles in mitigating pandemic and interpandemic influenza.

The high prevalence of self-reported preventive behavior use observed in this study demonstrates that the U.S. adult population are aware of and adopting some, if not most of these preventive behaviors to avoid catching influenza or to avoid spreading influenza to others. Though research has shown that these simple measures are highly effective in reducing virus transmission [19,23], some behavioral interventions are generally perceived as intrusive and/or different from typical day-to-day behavior (such as use of masks and gloves). Consequently, parents and teachers generally see these interventions as unacceptable in the context of seasonal influenza. Therefore, it has been suggested to target general etiquette practices (such as covering one’s cough and washing hands), as they are perceived as normal (by parents) and acceptable (by teachers) [24].

Through the use of multivariable modeling in our study, we found that several characteristics were independently associated with either higher or lower likelihood of adoption of the preventive behaviors studied. We observed that older adults, with a higher educational level, and from middle-higher income settings were more likely to adopt all or most of the behaviors than younger adults, those who were less educated, and those in a low-income setting. Similar results were reported [25] where older adults with a higher educational level and higher socioeconomic status were more likely to know that washing their hands with soap before and after touching raw poultry meat and using gloves were hygienic practices to avoid spreading the avian influenza virus through food. A significant association was also reported between those who fail to wash hands and use gloves and the lack of knowledge that these are standard hygienic practices to avoid spreading the avian influenza virus through food [25].

A systematic review [26] reported significantly higher reduction of respiratory infection among people in low/middle-income setting along with variation in the effectiveness of hand hygiene intervention. For adults, it has been suggested that a real or perceived financial or social disincentive might not allow them to stay home when sick with an infectious disease [24]. This finding might help explain the difference in adoption of preventive behaviors observed in this study among adults based on income settings. The results of our study further suggest that education and affordability might play important roles in personal and social behavior choices adults make to minimize risk of infectious disease transmission to and from themselves. It also underscores the importance of tailored educational and promotional strategies for dissemination and widespread adoption of preventive measures and implementation of public health policies [25]. Coordinated social marketing campaigns rather than single-strategy communication campaigns, going beyond health messages alone, have been reported to motivate behavior change to reduce the spread of infectious disease on a university campus. This is stated with the caveat that these changes are sometimes difficult to achieve in community or population-based campaigns [27].

We observed in our study that females were more likely than males to adopt each preventive behavior studied. Gender differences in washing hands with soap before and after touching potentially infective material or surfaces and use of gloves to prevent influenza transmission have been previously reported [25].

Beyond vaccination, the addition of preventive behaviors might be a critical tool to help reduce or slow the spread of influenza. Despite national awareness campaign efforts, influenza vaccination promotion activities, and events undertaken by CDC to educate the general population about the importance of influenza vaccination in collaboration with national and grassroots partner organizations [3,28,29], this study like others [17] found that non-Hispanic black and Hispanic adults are less likely to get the seasonal influenza vaccination as a preventive behavior. This finding suggests that hesitancy in influenza vaccine acceptance and uptake still exists among minority race/ethnicities and underscores the fact that continued and sustained efforts are needed to increase uptake of influenza vaccination by addressing underlying differences in motivation and barriers across racial/ethnic populations [17].

The findings are subject to several limitations. First, responses to the survey might be subject to recall bias because they were collected by self-report and vaccination was not verified by medical records. However, self-reported influenza vaccination status among adults has been shown to be sensitive and specific [30–33]. Second, the reported preventive behaviors might not necessarily equate to the performance of such behaviors. Whether or not the respondent actually performed the behavior(s) was not (could not be) validated by observation. Thus, responses might be subject to social-desirability bias. Third, the sample was based on respondents who self-select to participate in the Internet panel and agree to participate in the NIFS through an invitation that references influenza vaccination. Estimates obtained from this study might be biased if the participation processes (panel and NIFS) were related to receipt of vaccination. This may not be corrected through weighting. Fourth, the NIFS survey was conducted in English only, which might have resulted in nonrepresentation of those who speak other languages [17]. Fifth, because the sample was limited to non-institutionalized civilian adults, generalization is not possible beyond this population. Sixth, we observed that, among those vaccinated, only 89% said that vaccination is a behavior they use to avoid catching influenza. Among the unvaccinated, about three-fourths said vaccination is a preventive behavior they use; however, respondents indicated they were not vaccinated. This discrepancy could at least partially be explained because the survey was conducted early in the influenza season and these participants might have planned to get vaccinated later in the season. Seventh, we conducted a bias analysis to determine the influence of nonresponse in the results using demographic and geographic information available in the KnowledgePanel^®^ sampling frame. Tests for the smallest race/ethnicity group (non-Hispanic other/multiple category) showed the highest detectable levels of bias, most falling below substantively meaningful levels of 10 percentage points, indicating that release of additional sample might be needed during the conduct of the NIFS and changes to the weighting methods. Eighth, none of the behaviors to avoid catching influenza or to avoid spreading influenza were operationally defined to the participants taking the survey. Therefore, substantial variation in the interpreted meaning of these behaviors by the respondents could exist. Finally, though we controlled for some known variables in this study, there might be unknown confounders that were not controlled for that might have biased the study estimates, the extent of which is hard to estimate.

## Conclusions

The findings from this study identify the frequency with which recommended preventive behaviors to avoid catching influenza or spreading influenza to others have been adopted by adults in the United States.

Identifying the current usage of these preventive behaviors provides public health officials with critical information that can be used to tailor interventions to help reduce or slow influenza transmission. Further research is needed to better understand the role and impact of preventive behaviors adopted by adults on influenza transmission, and to better understand the motivation and/or reasons for choosing particular preventive behavior(s) over other(s).
